# The Effect of Thermal Stress on the Bacterial Microbiome of *Exaiptasia diaphana*

**DOI:** 10.3390/microorganisms8010020

**Published:** 2019-12-20

**Authors:** Leon M. Hartman, Madeleine J. H. van Oppen, Linda L. Blackall

**Affiliations:** 1 Department of Chemistry and Biotechnology, Swinburne University of Technology, John St, Hawthorn, Victoria 3122, Australia; 2 School of Biosciences, The University of Melbourne, Grattan St, Parkville, Victoria 3010, Australia; madeleine.van@unimelb.edu.au (M.J.H.v.O.); linda.blackall@unimelb.edu.au (L.L.B.); 3 Australian Institute of Marine Science, 1526 Cape Cleveland Rd, Cape Cleveland, Queensland 4810, Australia

**Keywords:** *Exaiptasia diaphana*, *Exaiptasia pallida*, thermal stress, microbiome, bacteria, coral model

## Abstract

Coral bleaching linked to climate change has generated interest in the response of coral’s bacterial microbiome to thermal stress. The sea anemone, *Exaiptasia diaphana*, is a popular coral model, but the response of its bacteria to thermal stress has been barely explored. To address this, we compared the bacterial communities of Great Barrier Reef (GBR) *E. diaphana* maintained at 26 °C or exposed to increasing temperature (26–33 °C) over two weeks. Communities were analyzed by metabarcoding of the bacterial 16S rRNA gene. Bleaching and Symbiodiniaceae health were assessed by Symbiodiniaceae cell density and dark-adapted quantum yield (F_v_/F_m_), respectively. Significant bleaching and reductions in F_v_/F_m_ occurred in the heat-treated anemones above 29 °C. Overall declines in bacterial alpha diversity in all anemones were also observed. Signs of bacterial change emerged above 31 °C. Some initial outcomes may have been influenced by relocation or starvation, but collectively, the bacterial community and taxa-level data suggested that heat was the primary driver of change above 32 °C. Six bacterial indicator species were identified as potential biomarkers for thermal stress. We conclude that the bacterial microbiome of GBR *E. diaphana* is generally stable until a thermal threshold is surpassed, after which significant changes occur.

## 1. Introduction

One of the most notable manifestations of human-induced climate change has been an increase in sea surface temperature (SST) [1]. The effect of elevated temperature on reef-building corals that live close to their thermal limit has been catastrophic, causing mass bleaching events worldwide [2,3]. Bleaching, wherein corals lose the intracellular algae (Symbiodiniaceae) that provide the majority of their nutrition through photosynthesis, typically leads to starvation and death of the host animal [4]. The subsequent loss of coral cover rapidly converts previously productive reef systems into patchy remnants or marine deserts, particularly when bleaching has occurred on a mass scale [5].

The coral holobiont comprises the host and its intracellular Symbiodiniaceae, as well as prokaryotes, viruses and fungi [6], which all contribute to host health and resilience, for example through nutrient provisioning and pathogen protection [7]. Therefore, investigating how corals’ bacterial communities change in response to elevated SST can help us understand the role they play in host survival [8].

Studies of coral-associated bacteria have found that communities can be highly dynamic and may change with seasonal temperature shifts [9,10]. Changes seemingly predictive of heat stress survival have also been recorded [11]. However, understanding the influence of bacteria on coral bleaching has been difficult as all combinations of bacterial community stability or change, and bleaching resistance or susceptibility during thermal stress have been observed [12,13,14]. This highlights the need for controlled, laboratory-based experiments to clarify the relationship between temperature-related bacterial community shifts and bleaching in cnidarians [15,16,17].

The sea anemone, *Exaiptasia diaphana*, is a much-used model for coral symbiosis studies [18,19]. Its ability to propagate asexually for rapid growth of clonal populations, basic maintenance requirements and coral-like bleaching response to environmental stressors have seen it widely adopted by the research community and several clonal lines of different geographic origin and algal symbiont type have been established. However, studies using *E. diaphana* to explore cnidarian responses to heat stress have focused largely on aspects of the host-Symbiodiniaceae relationship [20,21,22,23,24,25], whilst its bacterial microbiome has been almost wholly neglected and data from only two studies are available.

In a 2010 Master’s thesis, *E. diaphana* of unspecified origin were exposed to temperature ramped from 26 °C to 31 °C over ten days, then held at 31 °C for four days [26]. No significant differences between the associated bacterial communities of control and treated anemones were detected across the study period. However, poor resolution of chosen molecular biology methods negatively impacted the findings. In addition, exposing the anemones to a maximum of 31 °C meant the anemones may not have been thermally stressed, and no measurements of algal cell density or photosynthetic performance were taken to assess their condition. Although the study revealed few insights, it is acknowledged as the first investigation of heat-related changes in the *E. diaphana* microbiome.

More recently, differences between the bacterial associates of three *E. diaphana* clonal lines maintained at 32 °C for >2 years or grown at 25 °C were reported [27]. Four *E. diaphana*-Symbiodiniaceae pairings were analysed: CC7 of Atlantic Ocean origin harboring *Symbiodinium linucheae* or *Breviolum minutum*, H2 of Pacific Ocean origin harboring *Breviolum minutum*, and RS of Red Sea origin harboring *Symbiodinium microadriaticum*. Significant differences in some alpha diversity indices suggested higher bacterial community richness and lower evenness in the anemones grown at 32 °C and referred to by the authors as “heat stressed”. Notably, bacterial beta diversity and variability was higher in the heat-exposed anemones, apparently providing an example of the Anna Karenina Principle wherein microbiomes from healthy host species are similar, but those from unhealthy hosts are dysbiotic in their own way [28]. However, no evidence of thermal stress was provided and long-term maintenance of the anemones at 32 °C suggests they had become acclimated, particularly as the authors described them as having “reached a final stable state”. Therefore, the bacterial associates of the anemones at 32 °C likely exhibited an adjustment, albeit an inconsistent one, of the host rather than a response to temperature increase as would occur during a natural summer heatwave. This suggests that the response of *E. diaphana*’s bacterial associates to environmentally relevant heat stress remains unexplored. The present study addressed this knowledge gap by investigating bacterial changes in *E. diaphana*, originally sourced from Australia’s Great Barrier Reef (GBR), under thermal stress conditions comparable to those found in nature.

## 2. Materials and Methods

### 2.1. Experimental Conditions and Sample Processing

**Figure 1:**
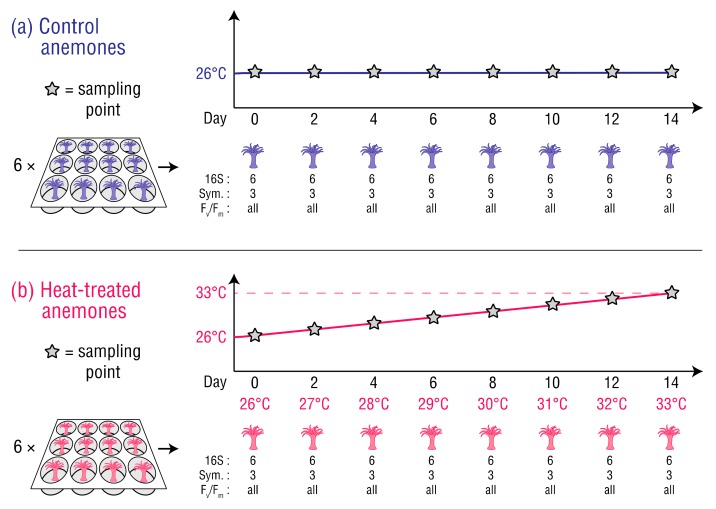
Sampling schedule for the (**a**) control anemones, and (**b**) heat-treated anemones. The number of anemones sampled at each timepoint is listed for metabarcoding of the 16S rRNA genes (16S), Symbiodiniaceae cell counts (Sym), and iPAM measurements (F_v_/F_m_).

Clonal GBR origin *E. diaphana* (genotype = AIMS2; *n* = 144) harbouring their natural symbiont, *B. minutum*, were randomly selected from a single tank in the University of Melbourne (UoM) culture collection. Genotyping and symbiont identification for these cultures has been previously described [29]. The selected anemones were individually relocated into single wells within sterile 12-well plates (Costar 3513, Corning, NY, USA), and placed in two Hi-Point 740 incubators, each with lighting intensity and spectra matching the culture collection. The anemones were then acclimated for 10 days at 26 °C in autoclaved seawater reconstituted from Red Sea Salt™ (hereafter, ‘RSS-water’) at a salinity of 34 parts per thousand and fed freshly hatched *Artemia salina* nauplii once during the acclimation period. The RSS-water was changed every two days. Lighting throughout the experiment was 31.8–33.8 µmol m^−2^ s^−1^ on a 12 h:12 h light–dark cycle. Following the 10 day acclimation period, 72 (heat-treated) anemones were exposed to temperature increasing from 26 °C to 33 °C over 14 days with a programmed increase of 0.5 °C per day, and 72 (control) anemones were maintained at 26 °C (Figure 1). Throughout the experiment, each incubator’s temperature was monitored by internal sensors, an independent electronic temperature probe and data logger (Saveris T3D, Testo, Lenzkirch, Germany), and glass thermometers (Initial, Brannan, Cleator Moor, England) placed in water-filled 500 mL Schott bottles. The anemones were not fed after acclimation to minimize the introduction of bacteria that could have contributed to their bacterial compositions. Starvation was considered reasonable as it is a common practice in *E. diaphana* studies [21,23,30,31,32].

On sampling days, the anemones were removed from the incubators 30–60 min after the daylight cycle and dark-adapted for 10 min before maximum quantum yield (F_v_/F_m_) was measured with an imaging pulse amplitude modulation (iPAM) fluorometer (IMAGING-PAM M-Series, Heinz Walz, Effeltrich, Germany). This allowed non-invasive monitoring of holobiont health by assessing damage to photosystem II (PSII) in the photosynthetic apparatus of *B. minutum* [33]. iPAM settings for all samples were: measuring light intensity 2, frequency 1; gain 2; damping 2. After each F_v_/F_m_ measurement, nine randomly selected control and heat-treated anemones were snap frozen in liquid nitrogen, three of each for Symbiodiniaceae density analysis to determine the extent of bleaching, and six of each for bacterial community analysis. All samples were stored at −80 °C until processing.

Anemones taken for Symbiodiniaceae density analysis were homogenized then centrifuged, and an aliquot of supernatant was removed for total protein measurement by Bradford Assay [34]. The pellet was washed twice and resuspended in filtered RSS-water. The Symbiodiniaceae cell density in the suspension was measured in triplicate on an automated cell counter (Life Technologies Countess II FL, Thermo Fisher, Scoresby, Australia), and values were normalized to total protein to account for anemone size differences. Sample DNA for bacterial analysis was extracted from the anemones following a protocol previously described [35] but modified with the inclusion of 15 min incubation with 20 mL of 10 mg/mL lysozyme after sample homogenization, and 20 s bead beating at 30 Hz (Tissue-Lyser II, Qiagen, Chadstone, Australia) with 100 mg of sterile glass beads (G8772, Sigma Aldrich, North Ryde, Australia). In preparation for DNA sequencing, sample DNA was amplified by PCR using primers with Illumina adapters (underlined) targeting the V5–V6 regions of the 16S rRNA gene *784F* (5′ TCGTCGGCAGCGTCAGATGTGTATAAGAGACAGAGGATTAGATACCCTGGTA 3′); *1061R* (5′ GTCTCGTGGGCTCGGAGATGTGTATAAGAGACAGCRRCACGAGCTGACGAC 3′) [36]. Triplicate PCRs were performed in 20 µL volumes of 1 µL template DNA, 10 µL MyTaq HS Mix polymerase (Bioline, Eveleigh, Australia), 0.5 µL of 10 µM *784F*, 0.5 µL of 10 µM *1061R*, and 8 µL MilliQ water. Thermal-cycler settings were: 1 cycle at 95.0 °C for 3 min, 30 cycles at 95.0 °C, 55.0 °C and 72.0 °C for 15 s each, and 1 cycle at 72 °C for 3 min. Each triplicate was pooled, then the product checked by 1% agarose gel electrophoresis. To identify contaminants introduced during sample preparation, blank DNA extractions and no-template PCRs were included as negative controls.

A volume of 25 µL of pooled PCR product from each sample, and three 25 µL aliquots of a 16 member mock community (Table S1), which was included to assess sample-sample sequencing consistency, were sent to the Ramaciotti Centre for Genomics (RCG), Sydney, Australia for sequencing on a single Illumina MiSeq v2 (2 × 250 bp) run. RCG performed PCR product clean-up and normalization as part of library preparation prior to sequencing. The resulting Illumina MiSeq data were deposited in the NCBI Sequence Read Archive under accession number PRJNA576764.

### 2.2. Sequencing Data Workflow

Raw, demultiplexed MiSeq reads were joined in QIIME2 v2018.4.0 [37]. Denoising, chimera filtering, and trimming was performed in DADA2 [38] to correct sequencing errors, remove primer sequences, and low quality bases. Amplicon sequence variants (ASVs) with one representative sequence were removed. Taxonomy was assigned in QIIME2 against a SILVA database (v 132) trained with a naïve Bayes classifier [39,40,41,42]. ASVs identified as eukaryotes, mitochondria, or chloroplasts were removed.

All subsequent data analyses were performed in R v3.6.0 [43] with differences considered significant at α = 0.05. Tabulated ASV counts, taxonomic assignments and metadata were imported into R and converted into a phyloseq object for ongoing analyses [44]. Rarefaction curves were generated in vegan [45] to assess whether the samples had been sequenced sufficiently to capture species diversity. Potential contaminants were identified using the ‘prevalence’ method in decontam [46] with the default threshold of *p* = 0.1. ASVs not present in every mock community sample were deemed contaminants and were removed from those samples. After removal of putative contaminants, stacked bar-charts describing the number of reads taxonomically assigned to class in the anemone samples, and species in the mock communities, were plotted with ggplot2 [47] to inspect bacterial community compositions.

### 2.3. Physiological and Microbiome Diversity Data Analyses

Average F_v_/F_m_ values and algal cell densities for the control and heat-treated samples were plotted over time. Overall changes were evaluated by repeated measures ANOVA after checking assumptions of normality by Shapiro–Wilk [48], and homogeneity of variance by Levene’s tests [49] with the R package car [50]. If significant differences between control and heat-treated data or a group-by-time interaction was detected, paired *t*-tests for each timepoint were performed to determine when differences occurred. Paired, or pairwise Student’s *t*-tests [51] with Benjamini–Hochberg correction [52], were also performed on the control and heat-treated data to identify when values differed significantly within each sample category. If data deviated from normality and/or homogeneity of variance, Kruskal–Wallis [53], Welch’s [54] or Mann–Whitney U tests [55] were performed.

Alpha diversity metrics for the anemone-associated bacterial communities were plotted against time after normalizing the count data by sub-sampling to 12,649 reads per sample. The number of observed ASVs was used as a measure of richness. Simpson index was used as a measure of community evenness [56]. Shannon index was calculated for assessment of overall alpha diversity [57]. Differences between the control and heat-treated anemones, and across time within each category, were assessed for statistical significance as described above. The number of co-occurring ASVs within control and heat-treated anemones between Day 0 and 14 of the study period were visualized in Venn diagrams [58] to assess bacterial community member transience.

To visualize changes in beta diversity of bacteria in the control and heat-treated anemones across time, an nMDS ordination was generated of all samples from Bray-Curtis dissimilarities of Hellinger transformed data. The ordination was separated by day to assist visual interpretation. Overall differences between the bacterial community compositions of the control and heat-treated anemones were assessed using Generalized Linear Models (GLM) of ASVs in the R package mvabund [59]. Analysis was performed on ASVs collapsed to genus, and the explanatory variables ‘treatment’ (i.e., control or heat-treated) and ‘time’. A negative binomial distribution was confirmed as appropriate for the data by visualization of the model residuals. Likelihood ratio tests (LRT) were used to determine the deviance (i.e., goodness of fit) of the competing models across 999 sampling iterations. As a treatment-by-time interaction was detected (Table S2), separate analyses were performed for each timepoint at the ASV level against ‘treatment’ to determine when significant differences between the bacterial communities of the control and heat-treated anemones occurred.

### 2.4. Analysis of Changes in Abundance of Selected Bacterial Taxa

Line plots of the six most abundant bacterial classes for the control and heat-treated anemones were generated to assess changes in community composition across the study period at a high taxonomic level. Bacteria of the genus *Vibrio* frequently cause disease in corals and *E. diaphana* at temperatures above 27 °C due to the upregulation of virulence factors [32,60]. Therefore, changes in the relative abundance of *Vibrio* ASVs were investigated to assess their prevalence, and hence disease-causing potential in GBR *E. diaphana* at elevated temperature. For the bacterial class-level and *Vibrio* analyses, significant differences between the control and heat-treated anemones, and across time within each category, were assessed as described above.

### 2.5. Indicator Species Identification

Individual taxa that differed significantly both in the heat-treated samples between Day 0 and Day 14, and between the control and heat-treated anemones at Day 14, were identified in an IndVal (Indicator Value) analysis implemented in the R package labdsv [61]. IndVal is recommended for discovering potential bacterial biomarkers in the microbiomes of corals subjected to environmental stressors [62], and has been used in previous coral research [63,64,65]. It combines specificity, defined as the mean abundance of a species within a sample type, and fidelity, defined as the relative frequency of occurrence of that species within sample types, to calculate the probability that the species discriminates between samples [66]. Relative abundances of IndVal-nominated taxa were plotted across the sampling timepoints to assess change.

## 3. Results

### 3.1. Sequencing Data and Bacterial Community Characteristics

**Figure 2:**
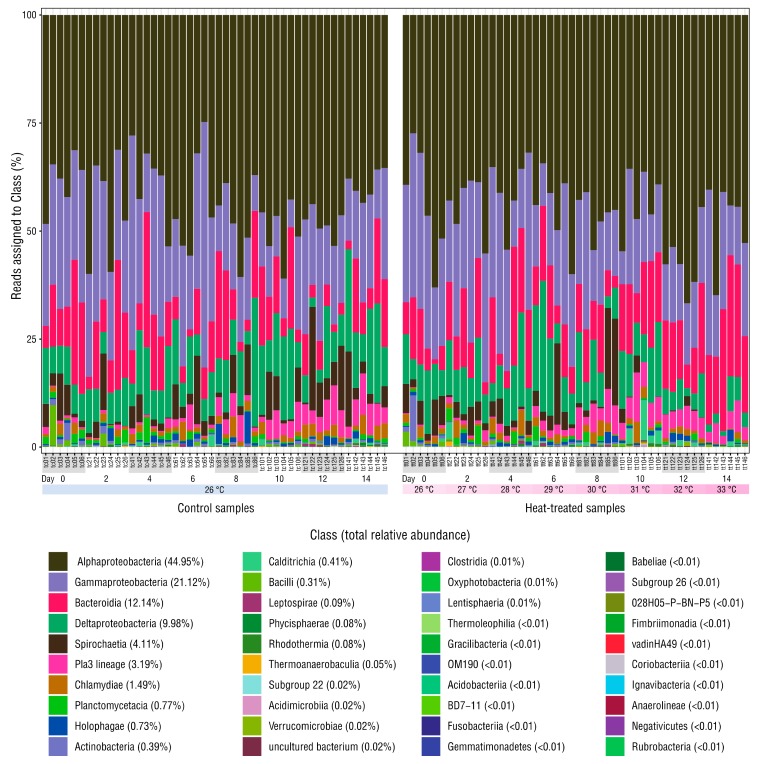
Relative abundance of reads assigned to each class. The mean relative abundance of each class is shown in brackets. Mock community and negative control samples are omitted.

Sequencing produced 4,543,989 raw reads across the 96 microbiome and three mock community samples: minimum 23,447; mean 45,899, maximum 70,071 reads per sample. After merging, denoising and chimera filtering, 2,972,541 reads remained (minimum 12,649, mean 30,026, maximum 46,995 reads per sample) and 4313 ASVs were identified. Rarefaction curves for all samples plateaued, indicating that sequencing captured bacterial diversity (Figure S1). Decontam removed eleven ASVs deemed contaminants, which constituted ~0.02% relative abundance of the bacterial communities in the anemone samples (Table S3). Compositions of the three replicate mock community samples were almost identical, indicating high sample–sample sequencing consistency (Figure S2). Stacked bar-charts of taxonomic classes detected in the anemones showed moderate variation in relative abundance in the samples with dominance by Alphaproteobacteria or Gammaproteobacteria taxa in most (Figure 2). Half of the 40 classes detected contained <0.02% relative abundance each.

### 3.2. Phenotypic Changes in the Anemones

**Figure 3:**
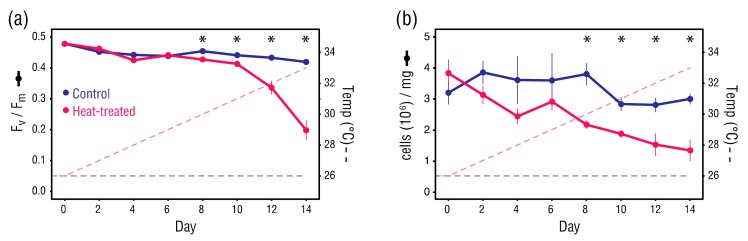
(**a**) Dark-adapted quantum yield (F_v_/F_m_) (at each datapoint, *n* = 6), and (**b**) Symbiodinaceae cell density (cells (10^6^)/mg) (at each datapoint, *n* = 3). Error bars ± 1 SEM. Asterisks indicate significant differences between control and heat-treated values (Student’s *t*-tests, α = 0.05).

Although the difference in dark-adapted quantum yield (F_v_/F_m_) of *B. minutum* between the control and heat-treated anemones was significant at Day 8 (Student’s *t*-test, *p* = 0.010) and Day 10 (Student’s *t*-test, *p* = 0.043), there was little difference in F_v_/F_m_ between the sample types until the temperature exceeded 31 °C on Day 10 (Figure 3a). Thereafter, F_v_/F_m_ values in the heat-treated anemones dropped sharply, clearly indicating the onset of damage to PSII in *B. minutum*, whereas F_v_/F_m_ values in the control anemones remained relatively stable.

Symbiodiniaceae cell densities in the heat-treated anemones declined steadily from Day 0, however values did not differ significantly from the control anemones until Day 8 (Student’s *t*-test, *p* = 0.013) (Figure 3b). Overall, Symbiodiniaceae cell density in the heat-treated anemones underwent a significant decline from 3.8 × 10^6^ to 1.3 × 10^6^ cells/mg host protein (Student’s *t*-test, *p* < 0.001). Although Symbiodinaceae cell density fluctuated in the control anemones, there was little difference between Day 0 and Day 14 values (3.2 × 10^6^ versus 3.0 × 10^6^ cells/mg host protein). The consistency of F_v_/F_m_ and cell densities in the control anemones throughout the treatment period suggest that Day 0 values approximate normal levels.

### 3.3. Changes in Alpha Diversity of the Bacterial Microbiomes

**Figure 4:**
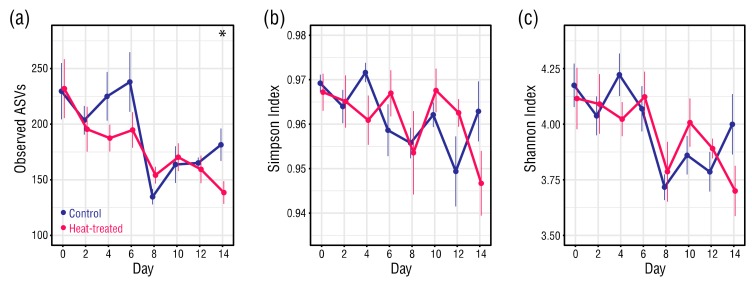
(**a**) Average number of observed amplicon sequence variants (ASVs), (**b**) Simpson index values, and (**c**) Shannon index values. At each datapoint, *n* = 6. Error bars ± 1 SEM. Asterisks indicate significant differences between control and heat-treated values (Student’s *t*-tests, α = 0.05).

Bacterial community richness fell significantly from Day 0 to Day 14 in both the control (Student’s *t*-test, *p* = 0.008) and heat-treated (Student’s *t*-test, *p* = 0.004) anemones (Figure 4a). At Day 0, the average number of bacterial ASVs in the control and heat-treated anemones was almost identical, but by Day 14 the bacterial ASVs in the control and heat-treated anemones had dropped on average to 181 and 139, respectively. Whilst the average number of bacterial ASVs in the heat-treated anemones followed a general downward trend, those in the control anemones almost halved between Day 6 (237) to Day 8 (135), before recovering. Despite their different trajectories, there was a significant difference in average observed ASVs between the control and heat-treated anemones only at Day 14 (Student’s *t*-test, *p* = 0.036). A survey of unique bacterial ASVs present in the control and heat-treated anemones at Days 0 or 14 further illustrated the initial similarity in richness in the sample types, and higher overall reduction in richness in the heat-treated anemones (Figure S3). Bacterial community evenness in the control and heat-treated anemones remained high and within a narrow range (Simpson index: 0.97–0.94) (Figure 4b). Although an overall drop in evenness for the heat-treated anemones was significant (Student’s *t*-test, *p* = 0.035), this did not result in a significant difference in Simpson evenness between the control and heat-treated anemones at Day 14. Shannon index values showed that overall alpha diversity of the bacteria in the control and heat-treated anemones fell throughout the treatment period, and the trend was generally comparable between the sample types (Figure 4c). Whilst the overall drop in Shannon diversity for the heat-treated anemone bacteria was significant (Student’s *t*-test, *p* = 0.042), the difference in Shannon diversity between the bacteria in the control and heat-treated anemones at Day 14 was not.

**Figure 5:**
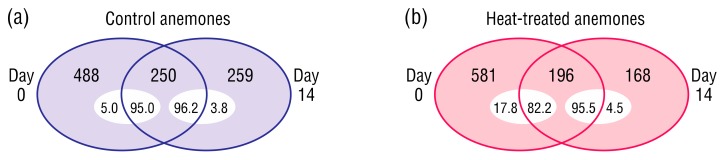
Unique and common bacterial ASVs at Day 0 and 14 in (**a**) control, or (**b**) heat-treated anemones. Inset numbers indicate relative abundance (%) on Day 0 or 14.

A survey of unique bacterial ASVs present in the control or heat-treated anemones at Days 0 and 14 provided an initial insight into changes in beta diversity within each sample type (Figure 5). Only a subset of ASVs in each sample type persisted to the end of the experiment, however they were the dominant community members (82.2–96.2% relative abundance). Bacterial ASVs detected at Day 14 but not Day 0 were likely low-abundance community members that increased to detectable levels, rather than bacteria introduced during the experiment, as the only input was sterile RSS-water.

### 3.4. Changes in Beta Diversity of the Bacterial Microbiomes

**Figure 6:**
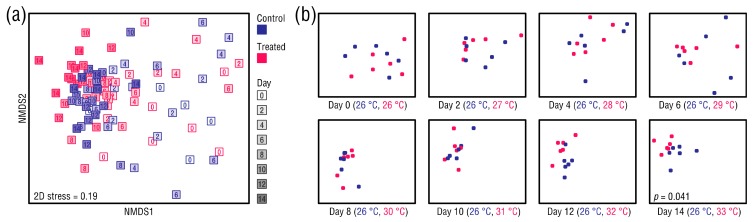
(**a**) nMDS ordination of the anemone-associated bacterial communities based on Bray–Curtis distances, and (**b**) plots showing datapoints from the original ordination for each day. Each datapoint represents one sampling unit.

Datapoints in an nMDS ordination of the anemone microbiomes converged during the study period, indicating that the bacterial communities of the control and heat-treated anemones became more similar over time (Figure 6a). Thus, time rather than treatment was the primary grouping factor. However, as seen in plots showing the original ordination separated by day (Figure 6b), the datapoints appeared to cluster in a group-wise manner at Day 12, suggesting that the control and heat-treated anemones were developing distinct bacterial community compositions. This trend continued through to Day 14, and a GLM analysis indicated that by Day 14 the bacterial communities of the control and heat-treated anemones had become significantly different (manyGLM, *p* = 0.041) (Table S4).

### 3.5. Changes in Abundance of Selected Bacterial Taxa

**Figure 7:**
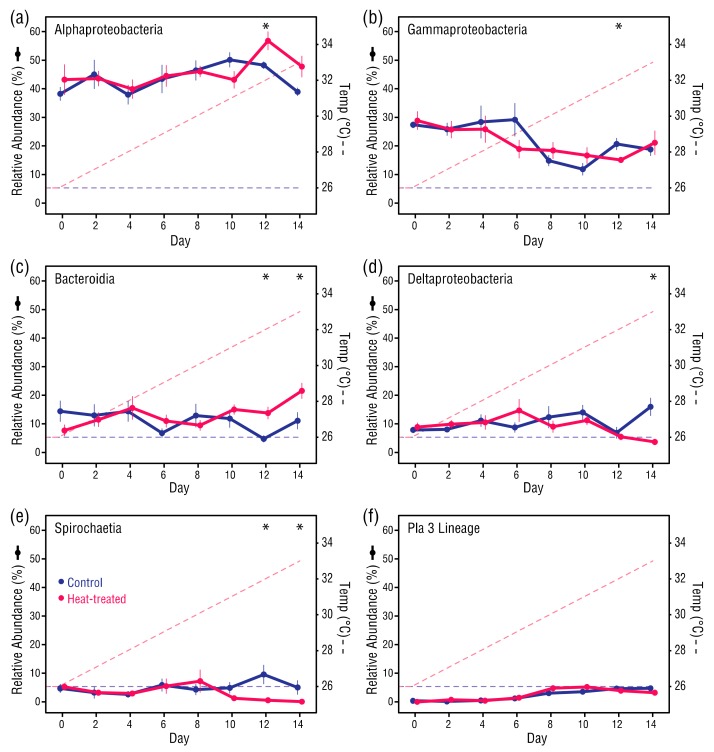
Changes in the six most abundant bacterial classes across all samples: (**a**) Alphaproteobacteria, (**b**) Gammaproteobacteria, (**c**) Bacteroidia, (**d**) Deltaproteobacteria, (**e**) Spirochaetia, and (**f**) Pla3 Lineage. For each datapoint, *n* = 6. Error bars ± 1SEM. Asterisks indicate significant differences between control and heat-treated values (see main text for test types, α = 0.05).

The six most abundant taxonomic classes across all samples were Alphaproteobacteria (44.95%), Gammaproteobacteria (21.12%), Bacteroidia (12.14%), Deltaproteobacteria (9.98%), Spirochaetia (4.1%) and Pla3 Lineage (3.2%), which accounted for >95% of all bacterial taxa (Figure 7). The relative abundance of most class-level bacterial taxa in control and heat-treated anemones was similar from Days 0 to 10. However, in some classes there was a divergence between the control and heat-treated anemones after Day 10, with significant increases in Alphaproteobacteria (Student’s *t*-test, *p* = 0.025) and Bacteroidia (Student’s *t*-test, *p* = 0.005), and decreases in Deltaproteobacteria (Welch’s *t*-test, *p* = 0.009) and Spirochaetia (Mann–Whitney U test, *p* = 0.031) in the heat-treated anemones compared to the control anemones. From Day 0 to Day 14, the control and heat-treated anemones underwent comparable decreases in Gammaproteobacteria taxa from 28.0% to 19.5%, although only the overall change in the control anemones was significant (Student’s *t*-test, *p* = 0.008), and comparable increases in Pla3 Lineage taxa from 0.6% to 5.0%, which was a significant increase in both the control (Mann–Whitney U test, *p* = 0.031) and heat-treated anemones (Mann–Whitney U test, *p* = 0.031).

**Figure 8:**
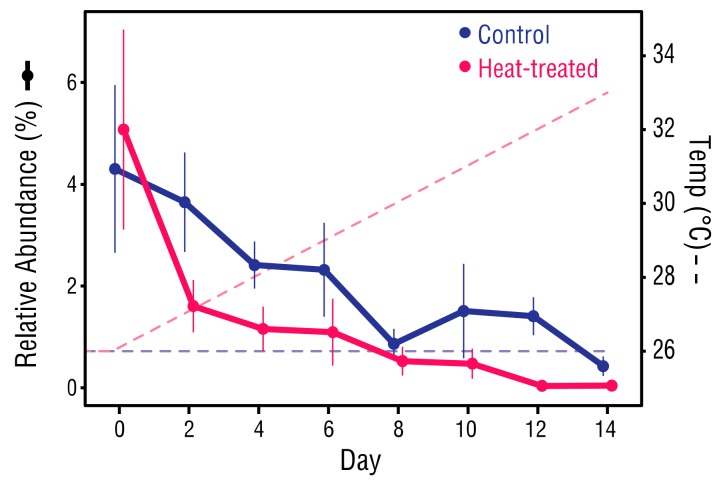
Changes in relative abundance of *Vibrio* sp. ASVs. For each datapoint, *n* = 6. Error bars ± 1SEM.

The bacterial communities of the control and heat-treated anemones experienced similar declines in ASVs of the genus *Vibrio* from Day 0, which generally continued throughout the study period. At Day 0, *Vibrio* averaged 4.7% of the bacteria in both control and heat-treated anemones (Figure 8). However, by Day 14, *Vibrio* had dropped significantly in the control anemones to 0.42% (Mann–Whitney U test, *p* = 0.031), and in the heat-treated anemones to 0.04% (Mann–Whitney U test, *p* = 0.031).

### 3.6. Indicator Species Identification

Twelve bacterial species were identified in an IndVal analysis (Table S5). However, only six showed changes in relative abundance suggestive of a response to elevated temperature (Figure S4). The others displayed high variability throughout the treatment period, making interpretation of their abundance changes difficult, and were thus discounted as potential indicator species (Figure S5). Of the six considered valid indicator species, an ASV from the family Saprospiraceae was moderately abundant in both the control and heat-treated anemones until Day 12, but from Day 12 to Day 14 it increased substantially in the heat-treated anemones from 4.9% to 13.3% relative abundance (Figure S4a). Two ASVs from the class Gammaproteobacteria and family Terasakiellaceae, respectively, were relatively stable until Days 8–10 (Figure S4b,c). Thereafter, both underwent rapid increases in relative abundance in the heat-treated anemones. A second Terasakiellaceae ASV increased in abundance in the heat-treated anemones compared to control anemones until Day 12, then dropped sharply; a pattern that was replicated to a lesser extent in the control anemones (Figure S4d). An indicator species of the genus *Spirochaeta* 2 (Figure S4e) was prevalent in both the control and heat-treated anemones at Day 0, averaging 4.9% relative abundance across all samples. Although the relative abundance of *Spirochaeta* 2 fluctuated in both groups, it dramatically decreased in the heat-treated anemones from Day 8 (7.1%) to Day 14 (0.1%). A sixth indicator species, from the family Rhizobiaceae (Figure S4f), also fluctuated but generally increased in abundance in the heat-treated anemones across the study period.

## 4. Discussion

### 4.1. Factors Underpinning Bleaching

*E. diaphana* that were exposed to rising temperature (26 °C to 33 °C) maintained near-normal F_v_/F_m_ values until 31 °C was exceeded, thus suggesting an upper thermal limit for quantum efficiency of photosystem II in the Symbiodiniaceae harbored by these anemones. However, a notable albeit non-significant drop in Symbiodiniaceae cell density as soon as the temperature increased above ambient, showed that the anemones had low resistance to thermal bleaching.

The rapid onset of bleaching in the heat-treated anemones may have been linked to lack of food. Starvation has been practiced in previous *E. diaphana* experiments [21,23,30,31,32] despite observed reductions in Symbiodiniaceae cell density following food deprivation [67,68,69], but the possible impact of this on experimental outcomes has rarely been acknowledged [70]. Our data suggest that, in future work, continued feeding is advisable, but at levels that maintain normal thermal tolerance rather than enhance it as seen in some coral species under heterotrophic conditions [71,72,73].

Although the incubator conditions were matched to the culture collection environment, relocation of the anemones may have also increased bleaching susceptibility as 10 days may have been inadequate for full acclimation. Consequently, longer acclimation periods may be advisable in studies with *E. diaphana* to avoid confounding.

### 4.2. Environmental Stressors Reduced Alpha Diversity

Overall, bacterial community richness decreased in the heat-treated anemones, which was contrary to previous reports [27]. However, such a comparison is difficult as the *E. diaphana* in [27] were held at 32 °C for >2 years and although referred to as ‘heat-stressed’, no evidence of this, such as reduced Symbiodiniaceae cell density compared to anemones at ambient temperature was provided, and no bleaching was reported. Reductions in bacterial alpha diversity have been seen in corals exposed to short-term [74] or long-term heat stress [13], and in other microbiomes subjected to environmental stressors, including heat [75]. Our results concur with these findings. However, increases in alpha diversity among heat-stressed corals are more common [76,77,78]. This may indicate that the behavior of *E. diaphana*’s bacterial microbiome is atypical among cnidarians, or that other factors influenced the bacterial community changes.

Shifts in bacterial alpha diversity in the control and heat-treated anemones were largely congruent, which could infer that heat was not, or was only partly responsible for changes in richness. The possible influence of starvation and incomplete acclimation on bleaching has been noted, with acclimation potentially playing a particular role in the initial changes seen in bacterial composition. For example, a study of corals transferred from a reef to aquaria found that the bacterial communities in the coral surface mucus layer (SML) took 14–28 days to stabilize [79]. Although the *E. diaphana* in the present study did not undergo such a dramatic relocation, the decline in richness among all the anemones could represent the late stages of acclimation, with the recovery in richness in the control anemones from Day 10 indicating a return to a normal state. As this return was not matched by the heat-treated anemones, it is reasonable to suggest that beyond Day 12 (i.e., above 32 °C) temperature was the main driver of change in alpha diversity for the heat-treated anemones.

### 4.3. Turnover of Low-Abundance ASVs Drive Shifts in Beta Diversity

Transience appears to be a common trait among coral and *E. diaphana* microbiome members [80,81,82], and this was evident in the present study as the majority of ASVs detected in the control and heat-stressed samples at Day 0 were not seen at Day 14, and vice versa (Figure 5). These transient bacteria, whilst comprising a small proportion of their bacterial communities (3.8–17.8%), were high in number, suggesting the presence of many species below the limit of detection that multiplied as conditions became favorable. Such a reservoir may benefit the host during stress by allowing their bacterial communities to restructure with members better at supporting holobiont homeostasis as proposed by the coral probiotic hypothesis [83].

The overall loss of richness in the control and heat-treated anemone bacterial associates likely led to the reduction in dissimilarity, and hence a reduction in beta diversity across the samples, by removing low-abundance bacteria that had inflated sample-sample dissimilarity. This is a common phenomenon [84]. However, differences in beta diversity emerged at Day 12 and became significant at Day 14, demonstrating that heat-stressed *E. diaphana* have bacterial associates distinct from *E. diaphana* at 26 °C. Further testing is needed to determine whether shifts induced by thermal stress stabilize, and whether the new bacterial communities can support the anemones for long periods at high temperature. Previous findings [27] suggest that high variability rather than uniformity eventuates in bacterial communities of *E. diaphana* exposed to high temperature. These changes may assist long-term survival at temperatures associated with bleaching in *E. diaphana* [22].

### 4.4. Changes in Bacterial Associates at a High Taxonomic Level were Apparent

The relative abundance of Alphaproteobacteria and Gammaproteobacteria in the control and heat-treated anemones was comparable up to 31 °C, then diverged significantly. However, the differences did not remain significant above 32 °C. Nevertheless, similar changes in these bacterial classes have been observed in thermally-stressed coral, which were attributed to shifts in the sugar composition of the coral SML [12]. Overall changes in relative abundance of some bacterial classes that were comparable in both control and heat-treated anemones, such as the decrease in Gammaproteobacteria and increase in Pla3 Lineage taxa, could be indicative of ongoing acclimation and normalization of the bacterial communities after relocation. However, relative increases in Bacteroidia and decreases in Deltaproteobacteria and Spirochaetia in the heat-treated anemones from Days 10–12, point to a temperature-influenced response and thermal tipping point for bacterial stability of 31–32 °C for GBR *E. diaphana*.

### 4.5. Were Vibrio Victims of Competition

The near elimination of *Vibrio* sp. from the heat treated anemone bacterial communities was unexpected as elevated temperature has been shown to increase *Vibrio* abundance in coral [78,85], posing a threat to coral health through temperature-induced upregulation of virulence factors [60,86]. Unexpected also was the parallel decline of *Vibrio* in the control and heat-treated anemones, which suggests factors common to all the bacterial associates, such as predation or antagonism by other bacteria, determined the fate of *Vibrio*. *Halobacteriovorax* sp. prey upon *Vibrio* [87] but none were detected. However, *Roseobacter* sp., which has members with antagonist activity against *Vibrio* sp. [88] were present in control and heat-treated anemones at all timepoints. Stressors common to both control and heat-treated anemones, relocation and nutrient deprivation, may have created a situation in which *Vibrio* were displaced by more competitive bacterial associate members [89]. Although possibly indicative of dysbiosis, the removal of potentially pathogenic *Vibrio* sp. could benefit thermally stressed *E. diaphana*.

### 4.6. Specific Bacteria as Biomarkers for Thermal Stress

Bacterial indicator species have been recommended as biomarkers for coral stress [62]. In the present study, the relative abundance of six bacterial species changed with rising temperature in ways that suggest they could be used to monitor the response of GBR *E. diaphana* exposed to thermal stress. The response of each indicator species might be due to temperature moving towards or away from a growth optimum, or other mechanisms.

An ASV from the family Saprospiraceae was the most abundant of the proposed indicator species. Saprospiraceae species have been shown to increase in abundance in heat-sensitive corals exposed to thermal stress [11]. Some prey on algae [90,91] or bacteria [92]. Therefore, the availability of released Symbiodiniaceae or an increase in bacterial prey in the heat-treated anemones could explain the increase of this ASV.

Two indicator species belonged to Terasakiellaceae, a bacterial family with members potentially involved in nitrogen cycling in some nutrient-limited corals [93]. Under ambient conditions, nitrogen availability is thought to be limited by cnidarian hosts to control Symbiodiniaceae division [94,95]. The increase of Terasakiellaceae ASVs may therefore signify opportunistic growth in a system where host-symbiont nitrogen regulation has been disrupted due to thermal stress.

An indicator species of the genus *Spirochaeta* 2 was almost eliminated in the heat-treated anemones above 30 °C, suggesting sensitivity to temperature. Bacteria from the phylum Spirochaetes have been identified in *E. diaphana* [82] and corals [96,97,98], including corals with high thermal tolerance [11]. However, the rapid decline of this indicator species above 30° C, shows it may serve as an early indicator of thermal stress in GBR *E. diaphana*.

The relative abundance of an indicator species from the family Rhizobiaceae increased as bleaching progressed. Bacteria in the family Rhizobiaceae may be intracellular associates of marine alga [99,100]. However, the increase of this ASV alongside a diminishing Symbiodiniaceae population may indicate that increasing temperature was a stronger promoter of growth than Symbiodiniaceae association. Despite this, it is interesting to note the possible relationship of several other indicator species with Symbiodiniaceae. Although the response of Symbiodiniaceae to temperature increase was rapid compared to the bacterial communities, thus suggesting the behavior of each was independent, it would be naïve to believe that the former might not impact the latter, either directly or through an overall impact on the holobiont.

Although a Gammaproteobacteria indicator species appeared to respond to temperature, lack of taxonomic identification limits speculation of its behavior. Another limitation of this and the other indicator species as universal biomarkers in future studies is a requirement for them to occur in the *E. diaphana* microbiomes. Due to the transience of many *E. diaphana* associated bacteria, this is uncertain. A further limitation, particularly for low-abundance species, is the use of relative rather than absolute abundance to describe changes in prevalence as this may skew abundances [101]. Bacterial load can be a strong indicator of stress or disease [102], and when used to transform count data into absolute abundance can provide a more comprehensive picture of microbiome dynamics [103]. Newly proposed methods such as spike-in of synthetic DNA during sample preparation could extend and improve microbiome data interpretability in future studies [104,105].

## 5. Conclusions

The bacterial microbiome of GBR *E. diaphana* is impacted by environmental stressors. In the present study, a reduction in bacterial community richness in the anemones at both ambient and elevated temperatures, and lowered bleaching resistance, may have been linked to incomplete acclimation or nutrient deprivation. However, differences between bacterial associates of control and heat-treated anemones in richness, beta diversity and taxon abundances that emerged above 31 °C and became significant above 32 °C suggest that temperature drives change above this threshold. Prolonged exposure to thermal stress may lead to further changes, such as increased beta diversity as proposed elsewhere, and this may support functions relevant to holobiont health. Some bacteria respond to thermal stress in ways that suggest they could be used to assess the impact of elevated temperature on GBR *E. diaphana.* These data improve our understanding of the *E. diaphana* bacterial microbiome, and hence this model organism’s utility in cnidarian bleaching research.

## Supplementary Materials

Supplementary materials can be found at https://www.mdpi.com/2076-2607/8/1/20/s1. Table S1: Mock community members Table S2: GLM analysis of differences in bacterial community beta diversity based on treatment (control vs. heat-treated) and time. Table S3: Putative contaminant ASVs removed from the dataset. Table S4: GLM analyses comparing bacterial community beta diversity at each sampling timepoint. Table S5: Potential indicator species identified in an IndVal analysis. Figure S1: Rarefaction curves for all samples. Figure S2: Relative abundance of reads assigned to genus in each mock community sample. Figure S3: Unique and common bacterial ASVs in control and heat-treated anemones at Day 0 or 14. Figure S4: ASVs identified by IndVal and considered potential indicator species. Figure S5: ASVs identified by IndVal but discounted as potential indicator species due to erratic changes in relative abundance and/or high variance.

## References

[1] Abram N.J., McGregor H.V., Tierney J.E., Evans M.N., McKay N.P., Kaufman D.S., Thirumalai K., Martrat B., Goosse H., Phipps S.J. (2016). Early onset of industrial-era warming across the oceans and continents. Nature.

[2] Coles S.L., Jokiel P.L., Lewis C.R. (1976). Thermal tolerance in tropical versus subtropical Pacific reef corals. Pac. Sci..

[3] Hughes T.P., Kerry J.T., Álvarez-Noriega M., Álvarez-Romero J.G., Anderson K.D., Baird A.H., Babcock R.C., Beger M., Bellwood D.R., Berkelmans R. (2017). Global warming and recurrent mass bleaching of corals. Nature.

[4] Tremblay P., Grover R., Maguer J., Legendre L., Ferrier-Pages C. (2012). Autotrophic carbon budget in coral tissue: A new C-13-based model of photosynthate translocation. J. Exp. Biol..

[5] Ostrander G.K., Armstrong K.M., Knobbe E.T., Gerace D., Scully E.P. (2000). Rapid transition in the structure of a coral reef community: The effects of coral bleaching and physical disturbance. Proc. Natl. Acad. Sci. USA.

[6] Rohwer F., Seguritan V., Azam F., Knowlton N. (2002). Diversity and distribution of coral-associated bacteria. Mar. Ecol. Prog. Ser..

[7] Krediet C.J., Ritchie K.B., Paul V.J., Teplitski M. (2013). Coral-associated micro-organisms and their roles in promoting coral health and thwarting diseases. Proc. R. Soc..

[8] Sharp K.H., Ritchie K.B. (2012). Multi-partner interactions in corals in the face of climate change. Biol. Bull..

[9] Cai L., Zhou G., Tong H., Tian R.-M., Zhang W., Ding W., Liu S., Huang H., Qian P.-Y. (2018). Season structures prokaryotic partners but not algal symbionts in subtropical hard corals. Appl. Microbiol. Biotechnol..

[10] Sharp K.H., Pratte Z.A., Kerwin A.H., Rotjan R.D., Stewart F.J. (2017). Season, but not symbiont state, drives microbiome structure in the temperate coral *Astrangia poculata*. Microbiome.

[11] Ziegler M., Seneca F.O., Yum L.K., Palumbi S.R., Voolstra C.R. (2017). Bacterial community dynamics are linked to patterns of coral heat tolerance. Nat. Commun..

[12] Lee S.T.M., Davy S.K., Tang S.-L., Kench P.S. (2016). Mucus sugar content shapes the bacterial community structure in thermally stressed *Acropora muricata*. Front. Microbiol..

[13] Tracy A.M., Koren O., Douglas N., Weil E., Harvell C.D. (2015). Persistent shifts in Caribbean coral microbiota are linked to the 2010 warm thermal anomaly. Environ. Microbiol. Rep..

[14] Gajigan A.P., Diaz L.A., Conaco C. (2017). Resilience of the prokaryotic microbial community of *Acropora digitifera* to elevated temperature. MicrobiologyOpen.

[15] Bourne D.G., Garren M., Work T.M., Rosenberg E., Smith G.W., Harvell C.D. (2009). Microbial disease and the coral holobiont. Trends Microbiol..

[16] Thompson J.R., Rivera H.E., Closek C.J., Medina M. (2015). Microbes in the coral holobiont: Partners through evolution, development, and ecological interactions. Front. Cell. Infect. Microbiol..

[17] Mouchka M.E., Hewson I., Harvell C.D. (2010). Coral-associated bacterial assemblages: Current knowledge and the potential for climate-driven impacts. Integr. Comp. Biol..

[18] Weis V.M., Davy S.K., Hoegh-Guldberg O., Rodriguez-Lanetty M., Pringle J.R. (2008). Cell biology in model systems as the key to understanding corals. Trends Ecol. Evol..

[19] Voolstra C.R. (2013). A journey into the wild of the cnidarian model system *Aiptasia* and its symbionts. Mol. Ecol..

[20] Tolleter D., Seneca F.O., DeNofrio J.C., Krediet C.J., Palumbi S.R., Pringle J.R., Grossman A.R. (2013). Coral bleaching independent of photosynthetic activity. Curr. Biol..

[21] Bieri T., Onishi M., Xiang T., Grossman A.R., Pringle J.R. (2016). Relative contributions of various cellular mechanisms to loss of algae during cnidarian bleaching. PLoS ONE.

[22] Gates R.D., Baghdasarian G., Muscatine L. (1992). Temperature stress causes host-cell detachment in symbiotic cnidarians: Implications for coral bleaching. Biol. Bull..

[23] Hillyer K.E., Tumanov S., Villas-Bôas S., Davy S.K. (2016). Metabolite profiling of symbiont and host during thermal stress and bleaching in a model cnidarian–dinoflagellate symbiosis. J. Exp. Biol..

[24] Núñez-Pons L., Bertocci I., Baghdasarian G. (2017). Symbiont dynamics during thermal acclimation using cnidarian-dinoflagellate model holobionts. Mar. Environ. Res..

[25] Gegner H.M., Ziegler M., Rädecker N., Buitrago-López C., Aranda M., Voolstra C.R. (2017). High salinity conveys thermotolerance in the coral model *Aiptasia*. Biol. Open.

[26] Plovie A. (2010). Comparison of Bacterial Communities Associated with Healthy and Bleached *Aiptasia pallida*, a Novel Model Organism for Coral Studies: Implications and Variation during Bleaching. Biochemistry and Biotechnology. Master’s Thesis.

[27] Ahmed H.I., Herrera M., Liew Y.J., Aranda M. (2019). Long-term temperature stress in the coral model *Aiptasia* supports the “Anna Karenina Principle” for bacterial microbiomes. Front. Microbiol..

[28] Zaneveld J.R., McMinds R., Vega Thurber R. (2017). Stress and stability: Applying the Anna Karenina principle to animal microbiomes. Nat. Microbiol..

[29] Dungan A.M., Hartman L.M., Tortorelli G., Belderock R., Lamb A.M., Pisan L., McFadden G., Blackall L.L., van Oppen M.J.H. (2019). *Exaiptasia diaphana* from the Great Barrier Reef: A valuable resource for coral symbiosis research. bioRxiv.

[30] Röthig T., Costa R.M., Simona F., Baumgarten S., Torres A.F., Radhakrishnan A., Aranda M., Voolstra C.R. (2016). Distinct bacterial communities associated with the coral model *Aiptasia* in aposymbiotic and symbiotic states with Symbiodinium. Front. Mar. Sci..

[31] Marty-Rivera M., Yudowski G., Roberson L. (2018). Mitigation of coral bleaching by antioxidants. bioRxiv.

[32] Zaragoza W.J., Krediet C.J., Meyer J.L., Canas G., Ritchie K.B., Teplitski M. (2014). Outcomes of infections of sea anemone *Aiptasia pallida* with *Vibrio* spp. pathogenic to corals. Microb. Ecol..

[33] Warner M.E., Fitt W.K., Schmidt G.W. (1999). Damage to photosystem II in symbiotic dinoflagellates: A determinant of coral bleaching. Proc. Natl. Acad. Sci. USA.

[34] Bradford M.M. (1976). A rapid and sensitive method for the quantitation of microgram quantities of protein utilizing the principle of protein-dye binding. Anal. Biochem..

[35] Wilson K., Li Y., Whan V., Lehnert S., Byrne K., Moore S., Pongsomboon S., Tassanakajon A., Rosenberg G., Ballment E. (2002). Genetic mapping of the black tiger shrimp *Penaeus monodon* with amplified fragment length polymorphism. Aquaculture.

[36] Andersson A.F., Lindberg M., Jakobsson H., Bäckhed F., Nyrén P., Engstrand L. (2008). Comparative analysis of human gut microbiota by barcoded pyrosequencing. PLoS ONE.

[37] Bolyen E., Rideout J.R., Dillon M.R., Bokulich N.A., Abnet C., Al-Ghalith G.A., Alexander H., Alm E.J., Arumugam M., Asnicar F. (2019). Reproducible, interactive, scalable and extensible microbiome data science using QIIME 2. Nat. Biotechnol..

[38] Callahan B.J., McMurdie P.J., Rosen M.J., Han A.W., Johnson A.J.A., Holmes S.P. (2016). DADA2: High-resolution sample inference from Illumina amplicon data. Nat. Methods.

[39] Wang Q., Garrith G.M., Tiedje J.M., Cole J.R. (2007). Naive bayesian classifier for rapid assignment of rRNA sequences into the new bacterial taxonomy. Appl. Environ. Microbiol..

[40] Pedregosa F., Varoquaux G., Gramfort A., Michel V., Thirion B., Grisel O., Blondel M., Prettenhofer P., Weiss R., Dubourg V. (2011). Scikit-learn: Machine learning in Python. J. Mach. Learn. Res..

[41] Quast C., Pruesse E., Yilmaz P., Gerken J., Schweer T., Yarza P., Peplies J., Glöckner F.O. (2013). The SILVA ribosomal RNA gene database project: Improved data processing and web-based tools. Nucleic Acids Res..

[42] Bokulich N.A., Kaehler B.D., Rideout J.R., Dillon M., Bolyen E., Knight R., Huttley G.A., Caporaso J.G. (2018). Optimizing taxonomic classification of marker-gene amplicon sequences with QIIME 2′s q2-feature-classifier plugin. Microbiome.

[43] R.C. Team (2018). R: A Language and Environment for Statisitical Computing.

[44] McMurdie P.J., Holmes S. (2013). Phyloseq: An R package for reproducible interactive analysis and graphics of microbiome census data. PLoS ONE.

[45] Oksanen J., Blanchet F.G., Kindt R., Legendre P., Minchin P.R., O’Hara R.B., Simpson G.L., Solymos P., Stevens M.H.H., Wagner H. Vegan: Community Ecology Package.

[46] Davis N.M., Proctor D.M., Holmes S.P., Relman D.A., Callahan B.J. (2018). Simple statistical identification and removal of contaminant sequences in marker-gene and metagenomics data. Microbiome.

[47] Wickham H. (2016). Ggplot2: Elegant Graphics for Data Analysis.

[48] Shapiro S.S., Wilk M.B. (1965). An analysis of variance test for normality complete samples. Biometrika.

[49] Levene H., Olkin I., Ghurye S.G., Hoeffding W., Madow W.G., Mann H.B. (1960). Robust tests for equality of variances. Contributions to Probability and Statistics: Essays in Honor of Harold Hotelling.

[50] Fox J., Weisberg S., Price B. Car: Companion to Applied Regression.

[51] (1908). Student. The probable error of a mean. Biometrika.

[52] Benjamini Y., Hochberg Y. (1995). Controlling the false discovery rate: A practical and powerful approach to multiple testing. J. R. Stat. Soc. Ser. B.

[53] Kruskal W.H., Wallis W.A. (1952). Use of ranks in one-criterion variance analysis. J. Am. Stat. Assoc..

[54] Welch B.L. (1947). The generalization of ‘Student’s’ problem when several different population variances are involved. Biometrika.

[55] Whitney J. (1997). Testing for differences with the nonparametric Mann–Whitney U test. J. Wound Ostomy Cont. Nurs..

[56] Simpson E.H. (1949). Measurement of diversity. Nature.

[57] Shannon C.E., Weaver W. (1949). The Mathematical Theory of Communication.

[58] Warnes G.R., Bolker B., Bonebakker L., Gentleman R., Huber W., Liaw A., Lumley T., Maechler M., Magnusson A., Moeller S. Gplots: Various R Programming Tools for Plotting Data.

[59] Wang Y., Naumann U., Wright S.T., Warton D.I. (2012). Mvabund—An R package for model-based analysis of multivariate abundance. Methods Ecol. Evol..

[60] Kimes N.E., Grim C.J., Johnson W.R., Hasan N.A., Tall B.D., Kothary M.H., Kiss H., Munk A.C., Tapia R., Green L. (2012). Temperature regulation of virulence factors in the pathogen *Vibrio Coralliilyticus*. ISME J..

[61] Roberts D.W. (2016). labdsv: Ordination and Multivariate Analysis for Ecology.

[62] Glasl B., Webster N.S., Bourne D.G. (2017). Microbial indicators as a diagnostic tool for assessing water quality and climate stress in coral reef ecosystems. Mar. Biol..

[63] Glasl B., Herndl G.J., Frade P.R. (2016). The microbiome of coral surface mucus has a key role in mediating holobiont health and survival upon disturbance. ISME J..

[64] Li J., Chen Q., Long L.-J., Dong J.-D., Yang J., Zhang S. (2014). Bacterial dynamics within the mucus, tissue and skeleton of the coral *Porites lutea* during different seasons. Sci. Rep..

[65] Astudillo-García C., Bell J.J., Webster N.S., Glasl B., Jompa J., Montoya J.M., Taylor M.W. (2017). Evaluating the core microbiota in complex communities: A systematic investigation. Environ. Microbiol..

[66] Dufrêne M., Legendre P. (1997). Species assemblages and indicator species: The need for a flexible asymmetrical approach. Ecol. Monogr..

[67] Clayton W.S., Lasker H.R. (1984). Host feeding regime and Zooxanthellal photosynthesis in the anemone, *Aiptasia pallida* (Verrill). Biol. Bull..

[68] Cook C.B., D’Elia C.F., Muller-Parker G. (1988). Host feeding and nutrient sufficiency for zooxanthellae in the sea anemone *Aiptasia pallida*. Mar. Biol..

[69] Davy S., Cook C. (2001). The relationship between nutritional status and carbon flux in the zooxanthellate sea anemone *Aiptasia pallida*. Mar. Biol..

[70] Lehnert E.M., Mouchka M.E., Burriesci M.S., Gallo N.D., Schwarz J.A., Pringle J.R. (2014). Extensive differences in gene expression between symbiotic and aposymbiotic cnidarians. G3.

[71] Grottoli A.G., Rodrigues L.J., Palardy J.E. (2006). Heterotrophic plasticity and resilience in bleached corals. Nature.

[72] Borell E.M., Yuliantri A.R., Bischof K., Richter C. (2008). The effect of heterotrophy on photosynthesis and tissue composition of two scleractinian corals under elevated temperature. J. Exp. Mar. Biol. Ecol..

[73] Ferrier-Pagès C., Rottier C., Beraud E., Levy O. (2010). Experimental assessment of the feeding effort of three scleractinian coral species during a thermal stress: Effect on the rates of photosynthesis. J. Exp. Mar. Biol. Ecol..

[74] Grottoli A.G., Dalcin Martins P., Wilkins M.J., Johnston M.D., Warner M.E., Cai W.-J., Melman T.F., Hoadley K.D., Pettay D.T., Levas S. (2018). Coral physiology and microbiome dynamics under combined warming and ocean acidification. PLoS ONE.

[75] Rocca J.D., Simonin M., Blaszczak J.R., Ernakovich J.G., Gibbons S.M., Midani F.S., Washburne A.D. (2019). The Microbiome Stress Project: Toward a global meta-analysis of environmental stressors and their effects on microbial communities. Front. Microbiol..

[76] Bourne D., Iida Y., Uthicke S., Smith-Keune C. (2008). Changes in coral-associated microbial communities during a bleaching event. ISME J..

[77] Santos H.F., Carmo F.L., Duarte G., Dini-Andreote F., Castro C.B., Rosado A.S., van Elsas J.D., Peixoto R.S. (2014). Climate change affects key nitrogen-fixing bacterial populations on coral reefs. ISME J..

[78] Tout J., Siboni N., Messer L.F., Garren M., Stocker R., Webster N.S., Ralph P.J., Seymour J.R. (2015). Increased seawater temperature increases the abundance and alters the structure of natural *Vibrio* populations associated with the coral *Pocillopora damicornis*. Front. Microbiol..

[79] Pratte Z.A., Richardson L.L., Mills D.K. (2015). Microbiota shifts in the surface mucopolysaccharide layer of corals transferred from natural to aquaria settings. J. Invertebr. Pathol..

[80] Hester E.R., Barott K.L., Nulton J., Vermeij M.J.A., Rohwer F.L. (2016). Stable and sporadic symbiotic communities of coral and algal holobionts. ISME J..

[81] Sweet M.J., Brown B.E., Dunne R.P., Singleton I., Bulling M. (2017). Evidence for rapid, tide-related shifts in the microbiome of the Coral *Coelastrea Aspera*. Coral Reefs.

[82] Brown T., Otero C., Grajales A., Rodríguez E., Rodriguez-Lanetty M. (2017). Worldwide exploration of the microbiome harbored by the cnidarian model, *Exaiptasia pallida* (Agassiz in Verrill, 1864) indicates a lack of bacterial association specificity at a lower taxonomic rank. PeerJ.

[83] Reshef L., Koren O., Loya Y., Zilber-Rosenberg I., Rosenberg E. (2006). The coral probiotic hypothesis. Environ. Microbiol..

[84] Shade A., Jones S.E., Caporaso J.G., Handelsman J., Knight R., Fierer N., Gilbert J.A. (2014). Conditionally rare taxa disproportionately contribute to temporal changes in microbial diversity. mBio.

[85] Vezzulli L., Previati M., Pruzzo C., Marchese A., Bourne D.G., Cerrano C. (2010). *Vibrio* infections triggering mass mortality events in a warming Mediterranean Sea. Environ. Microbiol..

[86] Munn C.B. (2015). The role of *Vibrios* in diseases of corals. Microbiol. Spectr..

[87] Welsh R.M., Zaneveld J.R., Rosales S.M., Payet J.P., Burkepile D.E., Thurber R.V. (2015). Bacterial predation in a marine host-associated microbiome. ISME J..

[88] Ruiz-Ponte C., Samain J.F., Sánchez J.L., Nicolas J.L. (1999). The benefit of a *Roseobacter* species on the survival of scallop larvae. Mar. Biotechnol..

[89] Hibbing M.E., Fuqua C., Parsek M.R., Peterson S.B. (2010). Bacterial competition: Surviving and thriving in the microbial jungle. Nat. Rev. Microbiol..

[90] Furusawa G., Yoshikawa T., Yasuda A., Sakata T. (2003). Algicidal activity and gliding motility of *Saprospira* sp. SS98-5. Can. J. Microbiol. Rev. Can. Microbiol..

[91] Shi M., Zou M., Liu X., Gao Y., Zhang Z., Wu W., Wen D., Chen Z., An C. (2006). A novel bacterium *Saprospira* sp. strain PdY3 forms bundles and lyses cyanobacteria. Front. Biosci..

[92] Lewin R.A. (1997). *Saprospira grandis*: A flexibacterium that can catch bacterial prey by ixotrophy. Microb. Ecol..

[93] Weiler B.A., Verhoeven J.T.P., Dufour S.C. (2018). Bacterial communities in tissues and surficial mucus of the cold-water coral *Paragorgia Arborea*. Front. Mar. Sci..

[94] Hoegh-Guldberg O., Smith G.J. (1989). Influence of the population density of zooxanthellae and supply of ammonium on the biomass and metabolic characteristics of the reef corals *Seriatopora hystrix* and *Stylophora pistillata*. Mar. Ecol. Prog. Ser..

[95] Muscatine L., Falkowski P.G., Dubinsky Z., Cook P.A., McCloskey L.R., Smith D.C. (1989). The effect of external nutrient resources on the population dynamics of zooxanthellae in a reef coral. Proc. R. Soc. B.

[96] Kimes N.E., Johnson W.R., Torralba M., Nelson K.E., Weil E., Morris P.J. (2013). The *Montastraea faveolata* microbiome: Ecological and temporal influences on a Caribbean reef-building coral in decline. Environ. Microbiol..

[97] Lawler S.N., Kellogg C.A., France S.C., Clostio R.W., Brooke S.D., Ross S.W. (2016). Coral-associated bacterial diversity is conserved across two deep-sea *Anthothela* species. Front. Microbiol..

[98] Closek C.J., Sunagawa S., DeSalvo M.K., Piceno Y.M., DeSantis T.Z., Brodie E.L., Weber M.X., Voolstra C.R., Andersen G.L., Medina M. (2014). Coral transcriptome and bacterial community profiles reveal distinct Yellow Band Disease states in *Orbicella faveolata*. ISME J..

[99] Hollants J., Leliaert F., Verbruggen H., Willems A., De Clerck O. (2013). Permanent residents or temporary lodgers: Characterizing intracellular bacterial communities in the siphonous green alga *Bryopsis*. Proc. R. Soc. B.

[100] Schwenk D., Nohynek L., Rischer H. (2014). Algae–bacteria association inferred by 16S rDNA similarity in established microalgae cultures. MicrobiologyOpen.

[101] Jackson D.A. (1997). Compositional data in community ecology: The paradigm or peril of proportions?. Ecology.

[102] Vandeputte D., Kathagen G., D’hoe K., Vieira-Silva S., Valles-Colomer M., Sabino J., Wang J., Tito R.Y., De Commer L., Darzi Y. (2017). Quantitative microbiome profiling links gut community variation to microbial load. Nature.

[103] Props R., Kerckhof F.M., Rubbens P., De Vrieze J., Hernandez Sanabria E., Waegeman W., Monsieurs P., Hammes F., Boon N. (2017). Absolute quantification of microbial taxon abundances. ISME J..

[104] Tourlousse D.M., Yoshiike S., Ohashi A., Matsukura S., Noda N., Sekiguchi Y. (2017). Synthetic spike-in standards for high-throughput 16S rRNA gene amplicon sequencing. Nucleic Acids Res..

[105] Stämmler F., Gläsner J., Hiergeist A., Holler E., Weber D., Oefner P.J., Gessner A., Spang R. (2016). Adjusting microbiome profiles for differences in microbial load by spike-in bacteria. Microbiome.

