# Supplementary material for: The Effect of Thermal Stress on the Bacterial Microbiome of Exaiptasia diaphana
Source: Microorganisms. 2019 Dec 20;8(1):20. doi: 10.3390/microorganisms8010020 (PMC7022623; doi:10.3390/microorganisms8010020)
Supplement: Supplementary file 1 [file microorganisms-08-00020-s001.pdf]

Table S1. Mock community members. The community was assembled from amplified near-complete 16S rRNA genes from the DNA of pure bacterial cultures. Taxonomic identification of community members was by Sanger sequencing of the 16S rRNA genes, then BLASTing manually trimmed sequences against the NCBI blastn database. Strain codes are in brackets.

1. *Alteromonas lipolytica* (MMFS00606)	2. *Bacillus pseudofirmus* (MMFS00610)
3. *Erythrobacter flavus* (MMFS00930)	4. *Exiguobacterium aestuarii* (MMFS00683)
5. *Labrenzia aggregata* (MMFS00933)	6. *Leisingera aquamixtae* (MMFS00623)
7. *Microbacterium hydrocarbonoxydans* (MMFS00614)	8. *Micrococcus aloeverae* (MMFS00068)
9. *Oceanicola litoreus* (MMFS00034)	10. *Pelagibaca bermudensis* (MMFS00670)
11. *Phaeobacter caeruleus* (MMFS00616)	12. *Pseudoalteromonas shioyasakiensis* (MMFS00019)
13. *Rhodococcus fascians* (MMFS006007)	14. *Tericoccus solisilvae* (MMFS00675)
15. *Thalassomonas ganghwensis* (MMFS00626)	16. *Vibrio alginolyticus* (MMFS00650)

Table S2: GLM analysis of differences in bacterial community beta diversity based on treatment (control vs heat-treated) and time.

Analysis of Deviance Table

Model: manyglm(formula = comboMva ~ combo$treatment * combo$time, family = "negative_binomial")

Multivariate test:

	Res.Df	Df.diff	Dev	Pr(>Dev)
(Intercept)	95			
combo$treatment	94	1	729	0.034 *
combo$time	87	7	5414	0.001 ***
combo$treatment:time	80	7	2610	0.001 ***

Signif. codes: 0 '***' 0.001 '**' 0.01 '*' 0.05 '. 0.1 ' ' 1. Arguments: Test statistics calculated assuming uncorrelated response (for faster computation); *p*-value calculated using 999 resampling iterations via PIT-trap resampling (to account for correlation in testing)

Table S3: Putative contaminant ASVs removed from the dataset.

	Phylum	Class	Order	Family	Genus	Relative abundance in anemone samples (%)	Relative abundance in mock community samples (%)
1	Proteobacteria	Alphaproteobacteria	Rhizobiales	Xanthobacteraceae	*Bradyrhizobium*	0.0002	0
2	Proteobacteria	Alphaproteobacteria	Rhizobiales	Rhizobiaceae	*Mesorhizobium*	<0.0001	0
3	Proteobacteria	Alphaproteobacteria	Rhizobiales	Xanthobacteraceae	*Afipia*	0.0002	0
4	Proteobacteria	Alphaproteobacteria	Sphingomonadales	Sphingomonadaceae	*Sphingomonas*	0.0051	0
5	Proteobacteria	Gammaproteobacteria	Betaproteobacteriales	Burkholderiaceae	*Pelomonas*	0.0003	0
6	Firmicutes	Bacilli	Lactobacillales	Enterococcaceae	*Enterococcus*	0.0025	0
7	Actinobacteria	Actinobacteria	Propionibacteriales	Propionibacteriaceae	*Cutibacterium*	0.0041	0
8	Bacteroidetes	Bacteroidia	Flavobacteriales	Flavobacteriaceae	NS3a marine group	0.0074	0
9	Bacteroidetes	Bacteroidia	Sphingobacteriales	Sphingobacteriaceae	*Sphingobacterium*	0.0001	0
10	Proteobacteria	Gammaproteobacteria	Betaproteobacteriales	Burkholderiaceae	*Burkholderia*	0.0015	0
11	Proteobacteria	Gammaproteobacteria	Betaproteobacteriales	Burkholderiaceae	*Ralstonia*	<0.0001	0
						Total (%):	Total (%):
						0.0214	0

Table S4: GLM analyses comparing bacterial community beta diversity at each sampling timepoint. Significant values are in bold (α = 0.05).

Day	Control (°C)	Heat-exposed (°C)	res.df	df	dev	*p*(>dev)	signif
0	26	26	10	1	1 725	0.156	
2	26	27	10	1	1 187	0.203	
4	26	28	10	1	1 417	0.181	
6	26	29	10	1	1 608	0.710	
8	26	30	10	1	805	0.147	
10	26	31	10	1	1 003	0.103	
12	26	32	10	1	1 103	0.052	.
14	26	33	10	1	1 217	**0.041**	*

Significance codes: 0 '***' 0.001 '**' 0.01 '*' 0.05 '.' 0.1 ' ' 1

p-value calculated using 999 sampling iterations.

Table S5: Potential indicator species identified in an IndVal analysis.

						Heat-treated anemones: Day 0 vs Day 14		Day 14 only: Control vs Heat-treated	
	Phylum	Class	Order	Family	Genus	IndVal stat.	*p*-value	IndVal stat.	*p*-value
1	Bacteroidetes	Bacteroidia	Chitinophagales	Saprospiraceae		0.8863684	0.003	0.7797039	0.016
2	Proteobacteria	Gammaproteobacteria				0.9878049	0.002	0.8766234	0.023
3	Proteobacteria	Alphaproteobacteria	Rhodospirillales	Terasakiellaceae		0.9589579	0.004	0.8659362	0.007
4	Proteobacteria	Alphaproteobacteria	Rhodospirillales	Terasakiellaceae		0.7888006	0.006	0.7036694	0.026
5	Spirochaetes	Spirochaetia	Spirochaetales	Spirochaetaceae	*Spirochaeta* 2	0.9972709	0.003	0.9977034	0.006
6	Proteobacteria	Alphaproteobacteria	Rhizobiales	Rhizobiaceae		0.8244275	0.004	0.6933535	0.012
7	Proteobacteria	Deltaproteobacteria	Oligoflexales	Oligoflexaceae		0.8023567	0.021	0.9661520	0.007
8	Planctomycetes	Planctomycetacia	Planctomycetales	Rubinisphaeraceae		0.7425532	0.034	0.8097484	0.036
9	Bacteroidetes	Bacteroidia	Chitinophagales	Saprospiraceae		0.9940728	0.004	0.9895366	0.003
10	Bacteroidetes	Bacteroidia	Chitinophagales			0.7589839	0.026	0.7792621	0.019
11	Proteobacteria	Deltaproteobacteria	Oligoflexales	Oligoflexaceae		0.8023567	0.021	0.8052620	0.018
12	Chlamydiae	Chlamydiae	Chlamydiales	Simkaniaceae		0.9413919	0.006	0.7793103	0.020

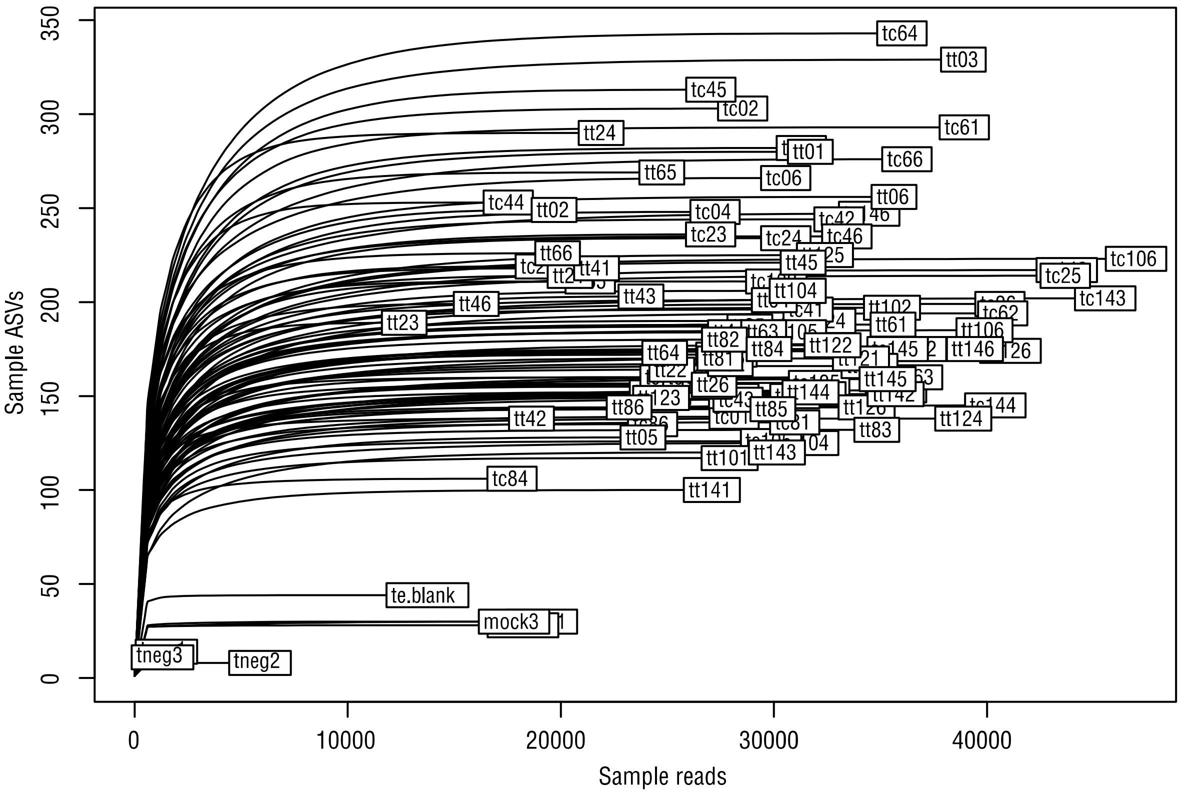

Figure S1: Rarefaction curves for all samples. Sub-sampling: 12,000; step: 600.

<u>Guide to sample names in Figure S1</u>

16S samples (e.g. tc125): first letter (t)= thermal stress experiment

second letter (c or t) = control or heat-treatment sample

first 1-2 digits (0-14) = sampling day 0-14

last digit = replicate no. 1-6

Negative PCR controls (e.g. tneg2):

first letter (t) = thermal stress experiment + neg + replicate no. (1-3)

Mock community samples (e.g. mock2): mock + replicate no. (1-3)

DNA extraction negative control = te.blank

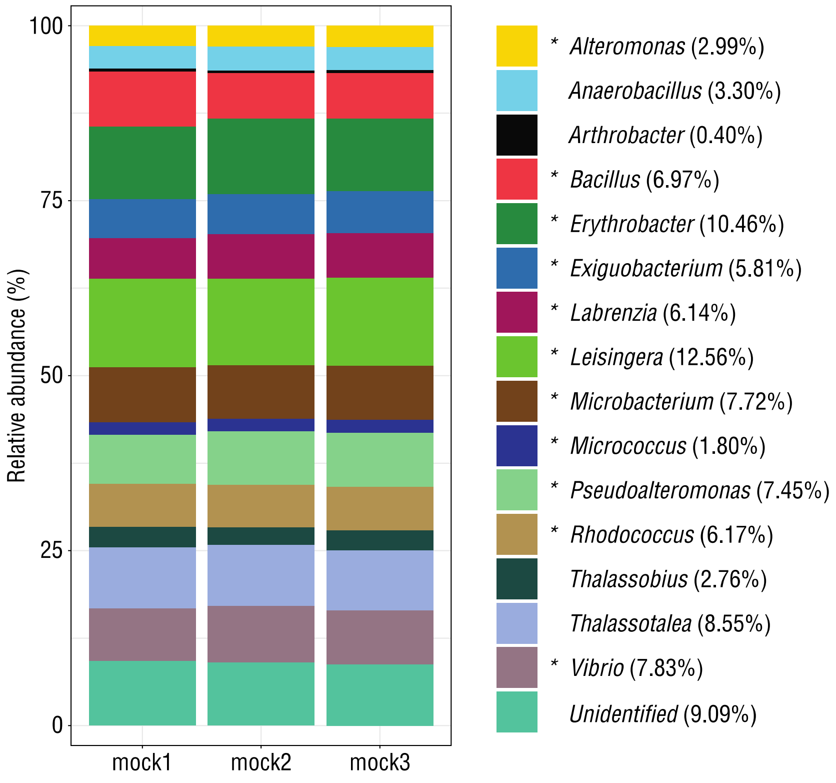

Figure S2: Relative abundance of reads assigned to genus in each mock community sample. Asterisks indicate genera present in the original mock community. Numbers in brackets are average relative abundances. 'Unidentified' contained three ASVs not identified to genus level.

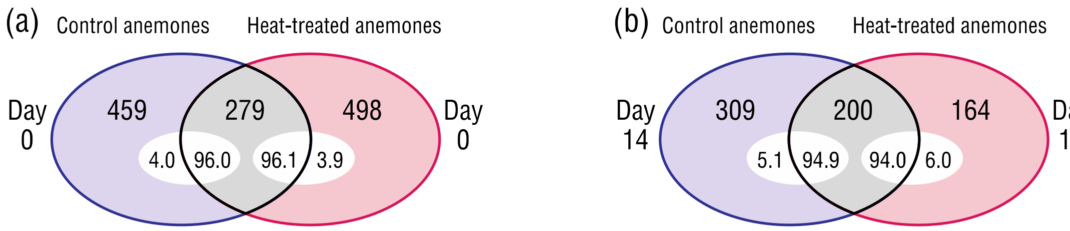

Figure S3: Unique and common bacterial ASVs in control and heat-treated anemones at Day 0 or 14. Inset numbers indicate relative abundance (%) for each sample type. Bacterial richness was highly similar at Day 0, with a high overlap in shared ASVs (**a**). Richness in both sample types declined throughout the treatment period, with the heat-treated anemones undergoing a more substantial drop by Day 14 (**b**). However, the number of shared ASVs remained high.

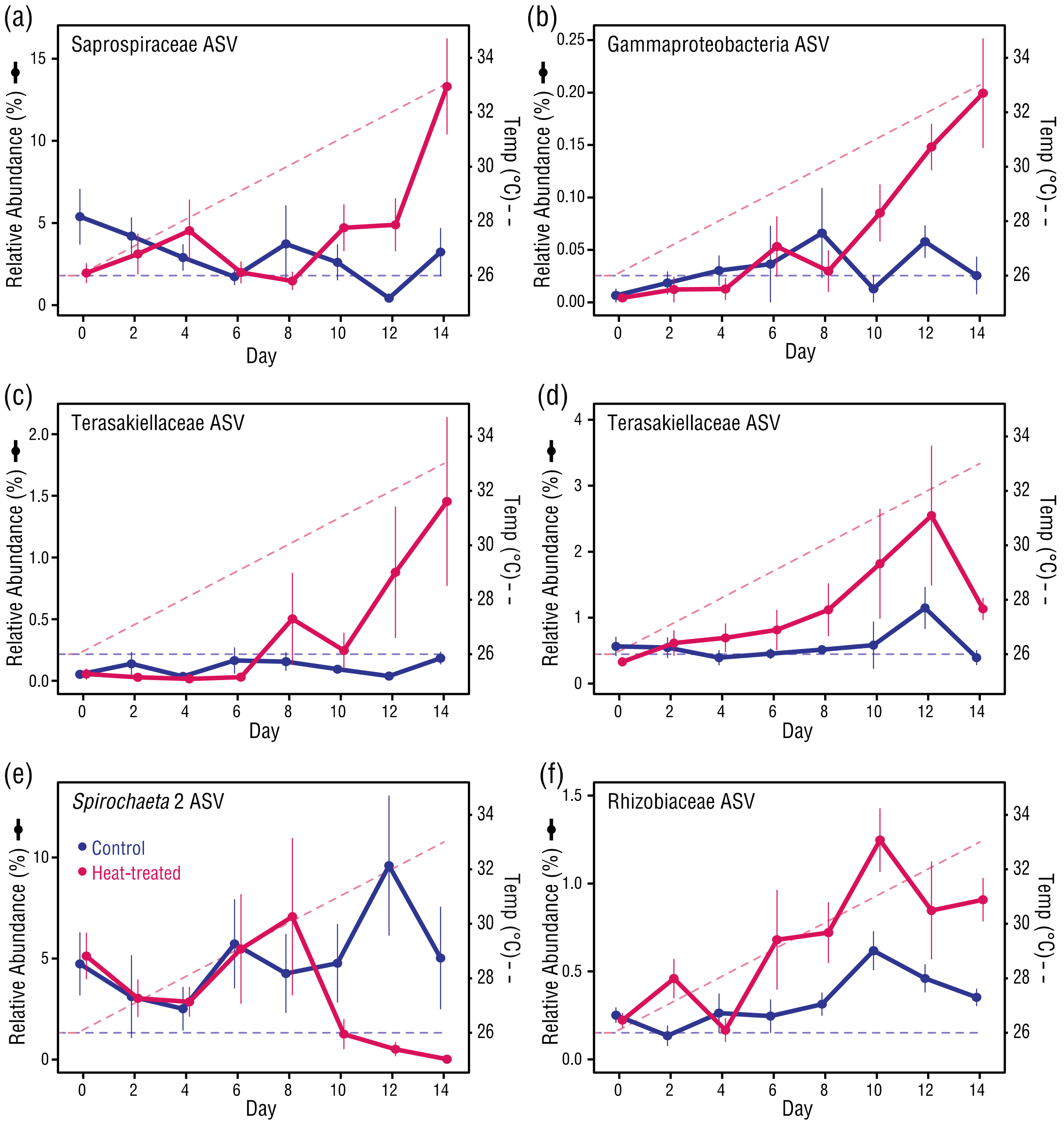

Figure S4: ASVs identified by IndVal and considered potential indicator species: (**a**) Saprospiraceae, (**b**) Gammaproteobacteria, (**c**) Terasakiellaceae, (**d**) Terasakiellaceae, (**e**) *Spirochaeta* 2, and (**f**) Rhizobiaceae. For each datapoint, $n = 6$. Error bars ± 1SEM.

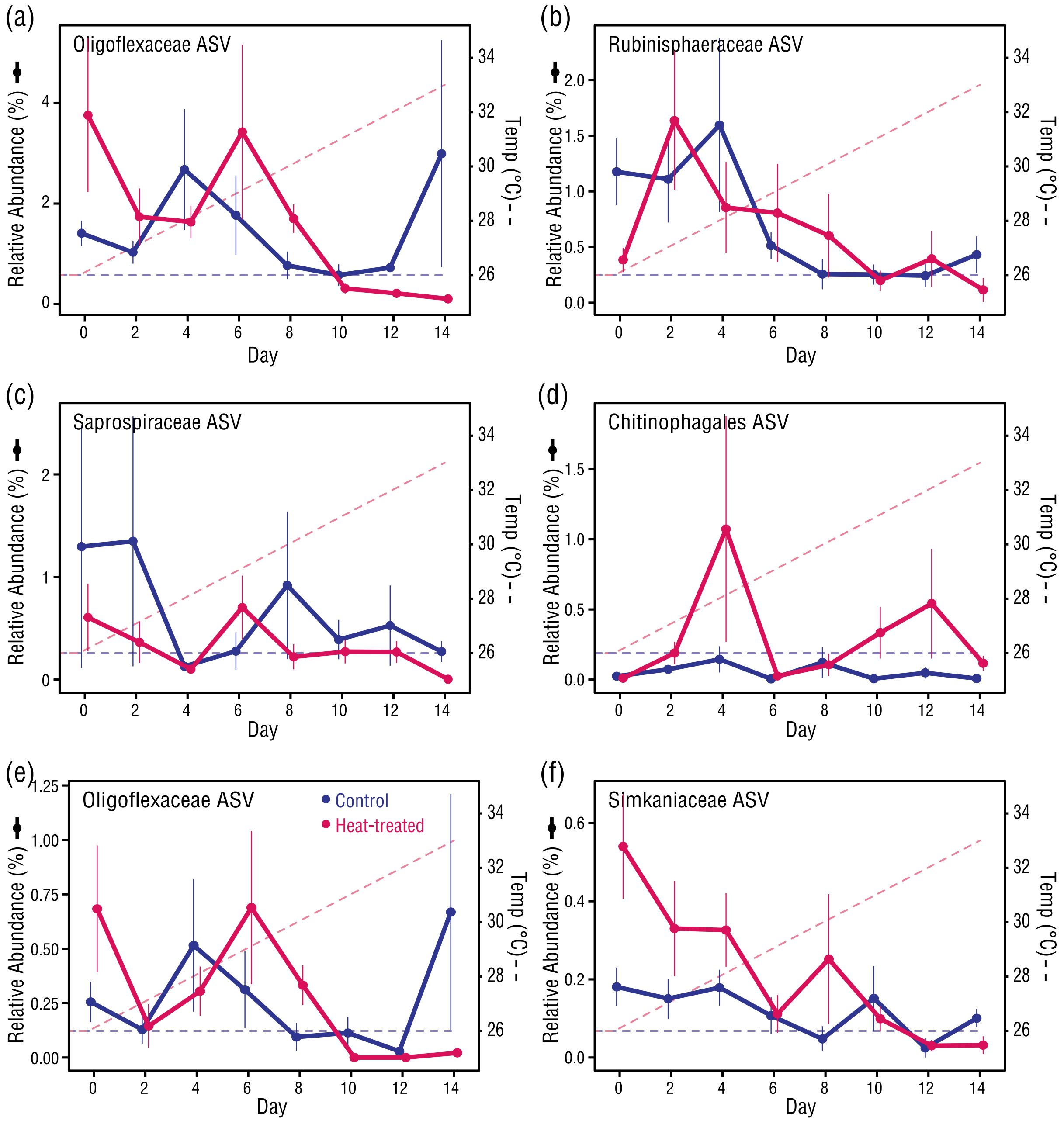

Figure S5: ASVs identified by IndVal but discounted as potential indicator species due to erratic changes in relative abundance and/or high variance. Simkaniaceae was discounted due to the large difference in relative abundance at Day 0 compared to all other timepoints: (**a**) Oligoflexaceae, (**b**) Rubinisphaeraceae, (**c**) Saprospiraceae, (**d**) Chitinophagales, (**e**) Oligoflexaceae, and (**f**) Simkaniaceae. For each datapoint, n = 6. Error bars ±1SEM.
